# Monitoring SARS-CoV-2 infection in different animal species and human in Egypt during 2020–2021

**DOI:** 10.1007/s11756-023-01362-1

**Published:** 2023-03-17

**Authors:** Mohammed A. AboElkhair, Mohamed M. Ahmed, Alaa El Din H. Moustapha, Ali Mohammed Zaki, Rania F. El Naggar, Moustafa Elhamouly, Anis Anis

**Affiliations:** 1 Department of Virology, Faculty of Veterinary Medicine, University of Sadat City, Sadat City, 32897 Egypt; 2 Department of Biochemistry and Chemistry of Nutrition, Faculty of Veterinary Medicine, University of Sadat City, Sadat City, 32897 Egypt; 3 Department of Bacteriology, Mycology and Immunology, Faculty of Veterinary Medicine, University of Sadat City, Sadat City, 32897 Egypt; 4 Department of Microbiology and Immunology, Faculty of Medicine, University of Ain-Shams, Cairo, 11591 Egypt; 5 Department of Histology and Cytology, Faculty of Veterinary Medicine, University of Sadat City, Sadat City, 32897 Egypt; 6 Department of Pathology, Faculty of Veterinary Medicine, University of Sadat City, Sadat City, 32897 Egypt

**Keywords:** Coronaviruses, SARS-CoV-2, RT-PCR, Animals, Egypt

## Abstract

Coronaviruses cause respiratory and intestinal infections in animals and humans. By the end of 2019, there was an epidemic of novel coronavirus (COVID-19), which is caused by the severe acute respiratory syndrome coronavirus 2 (SARS-CoV-2). Coronaviruses have a highly mutable genome that makes them genetically and phenotypically modifiable with a potential transmission to new host species. Based on current sequence databases, all human coronaviruses have animal origins, so animals have important roles in virus spillover to humans. The aim of this study is to investigate the role of different animal species in the epidemiology of SARS-CoV-2 in Egypt. A pan-coronaviruses RT-PCR has been used for detection of possible coronaviruses infection in different species including bats, humans, birds, and dogs in Egypt during the period of November 2020 till June 2021. Ninety-two samples (46 from *Rousettus aegyptiacus* bats, 10 from human, 26 from wild birds, and 10 from dogs) were screened for SARS-CoV-2. Our results revealed that only human samples were SARS-CoV-2 positive for SARS-CoV-2 while all other animal and bird samples were negative. To recapitulate, our results suggest that animals may not actively transmit SARS-CoV-2 among people in Egypt during the current COVID-19 pandemic. Further structural surveillance and follow up screening for SARS-CoV-2 among domestic and wild animal populations in Egypt is crucially needed.

## Introduction

Coronaviruses (CoVs) are enveloped viruses with positive sense, single-stranded RNA genome; belong to the subfamily *Orthocoronavirinae*, family *Coronaviridae*. *Orthocoronavirinae* has four genera alpha (α), beta (β), gamma (γ), and delta (δ) coronaviruses (Pal et al. 2020). CoVs have RNA genome that encodes structural, non-structural and accessory proteins. Structural proteins include spike, envelope, membrane, nucleocapsid and some viruses encode hemagglutinin-esterase (Fehr and Perlman 2015; Masters 2006). Among non-structural proteins, RNA dependent RNA polymerase (RdRP) contains the most conserved protein domain of all CoVs (Hu et al. 2018).

Mammals are the natural hosts of CoVs; bats are the natural host of α and β CoVs, while pigs and birds are natural hosts of γ and δ CoVs (Velavan and Meyer 2020). CoVs are diverse due to their ability to mutate (Woo et al. 2009) and its diversity is facilitated by the low proof reading of RdRP, high frequency of RNA recombination, and their large genomes (Forni et al. 2017; Su et al. 2016). These characters have led to the emergence of new viruses with new traits that are able to adapt to new hosts and ecologic niches, with their ability to spillover crossing the species barrier to infect humans resulting in epidemics (Woo et al. 2009).

Beta-coronaviruses include the three most infectious zoonotic viruses: SARS-CoV-1, MERS-CoV and SARS-CoV-2. SARS-CoV-2 has been classified as a novel emerging zoonotic member of subgenus sarbecovirus of the β- coronavirus. Although the bat-originated SARS-CoV-2 was proposed to have spread to people, there is still much information about the virus that has not been known, including how it moves between animals and people, and whether it has other hosts (Wang et al. 2020). Many models have been developed to evaluate the animal susceptibility to SARS-CoV-2 based on the implied affinity of the species’ angiotensin-converting enzyme 2 (ACE2) receptor-binding domain sites for the SARS-CoV-2 spike protein (Chen et al. 2020; Zhao et al. 2020). These studies classified animal’s susceptibility and their possible role as intermediate or reservoir host species (Damas et al. 2020). Moreover, the list of animal species susceptible to SARS-CoV-2 infection continues to grow. As of May 31, 2022, 676 outbreaks in animals have been reported globally, affecting 23 species in 36 countries (World Organization for Animal Health. SARS-CoV-2 in Animal- Situation Report 13, 2022). The vulnerability of diverse animal species to infection and the role of animals in the epidemiology of the present SARS-CoV-2 pandemic are crucial for understanding the current SARS-CoV-2 pandemic. Animals have not been subjected to broad testing or organized observation. Therefore, it is indispensable to identify the potential virus reservoir and the possibility of infection for other animal species. This study aims to provide an overview of the relation between SARS-CoV-2 and different animals in relation to humans in Egypt during the period from November 2020 to June 2021.

## Materials and methods

### Samples collections

The samples were collected from different Egyptian provinces during the period from November 2020 to June 2021 (Table 1). Bat sampling was performed by trained field personnel in Itay El Barud, Beheira province. No anesthetic or immobilization agents were used during capture. Rectal swabs were collected from every individual bat. Migratory bird cloacal swabs were collected from Common teal, northern Pintail and Mallard. Nasal swabs were collected from dogs in private pet clinics in Minoufiya province. All samples were placed into 500 μL TRIzol reagent and stored in a − 80 °C freezer until analysis. Human nasopharyngeal swabs were collected on viral transport medium under supervision of Dr. Ali Zaki, Professor of Microbiology, Faculty of Medicine, Ain Shams University and stored in a − 80 °C freezer. All animal handling procedures as well as samples’ collection and disposal were carried out according to the regulations of the Institutional Animal Care and Use Committee (IACUC), Faculty of Veterinary Medicine, University of Sadat City (Ethical approval number: VUSC-019-1-20).

**Table 1:** Samples used for the study

Species	Type of samples	No. of samples	Geographical location	Time period
Bats	Rectal swabs	46	Beheira province	November, 2020
Human	Nasopharyngeal swabs	10	Cairo province	January, 2021
Migratory birds	Cloacal swabs	20	Damietta province	February, 2021
Wild birds	Cloacal swabs	6	Minoufiya province	April, 2021
Pet animals	Nasal swabs	10	Minoufiya province	June, 2021

### RNA extraction and cDNA synthesis

Samples testings were performed at the Central Diagnostic Virology laboratory, Faculty of Veterinary Medicine, University of Sadat City. Swabs (human, bats, dogs, and birds) were used for RNA extraction. RNA was extracted using Direct-Zol RNA columns (Cat# R2050 Zymo Research Corp, CA92614, USA) following the manufacture instructions. cDNA synthesis was carried out using RevertAid First Strand cDNA Synthesis kit (Cat# K1622 Thermo Scientific, Dreiech, Germany) according to the manufacturer’s protocol. To ensure efficiency of RNA extraction from bats and human, internal control primer for GADPH (provided in RevertAid Kit) was used. Also, positive control infectious bronchitis vaccine (Combivac®, JOVAC, Amman, Jordan) was used for evaluation of RNA extraction kit and efficiency of employed primers.

### Amplification conditions for panCoVs PCR assay and sequencing

Initially, four sets of pan-coronaviruses primers (Macrogen, GAsa-dong, Geumcheon-gu, Korea) listed in (Table 2) were tested using positive control cDNA. Subsequently, samples cDNAs were screened for coronaviruses using a selected set of consensus primers (PanCoV- F2 and PanCoV- R1). The selected primer set targets a 668 bp- fragment of the RdRp of orf1ab of CoVs (Hu et al. 2018) (Table 2).

**Table 2:** Primer sets used for Pan-coronavirus RT-PCR

Oligonucleotide	Sequencing (5′-3′)	Amplicon (bP)	Reference
PanCoV- F2	AARTTYTAYGGHGGYTGG	668	Hu et al. (2018)
PanCoV- R1	GARCARAATTCATGHGGDCC		
Cor-FW	ACWCARHTVAAYYTNAARTAYGC	251	Vijgen et al. (2008)
Cor-RV	TCRCAYTTDGGRTARTCCCA		
IN-2	GGGTTGGGACTATCCTAAGTGTGA	452	Hasoksuz et al. (2007)
IN-4	TAACACACAACICCATCATCA		
Primer 1	GGTTGGGACTATCCTAAGTGTGA	440	Dominguez et al. (2007)
Primer 2	CCATCATCAGATAGAATCATCATA		

PCR was conducted in a final volume of 25 μL consisting of 3 μL cDNA, 1 μL of 10 picomolar of each primer (upstream and downstream), and 12.5 μl 2 × GoTaq® PCR master mix (Promega Corporation, Madison, WI). The final volume was made up to 25 μL using sterilized, nuclease-free deionized water. The PCR condition was carried out as previously described (Hu et al. 2018). Negative control in this assay was RNase-free water.

The PCR products were purified using the GeneJET Gel Extraction Kit (Thermo Scientific™, USA). Both strands of the PCR products were sequenced with an ABI Prism 3700 DNA analyzer (Applied Biosystems, Foster City, CA) by using the forward and reverse PCR primers. The obtained sequences were edited and analyzed using BioEdit V 7.1.3 (Hall 1999), and compared with known RdRp gene sequences of CoVs sequences in the database. The nucleotide sequences developed in the current study were submitted to the GISAID EpiCov database (http://www.gisaid.org).

## Results

**Fig. 1:**
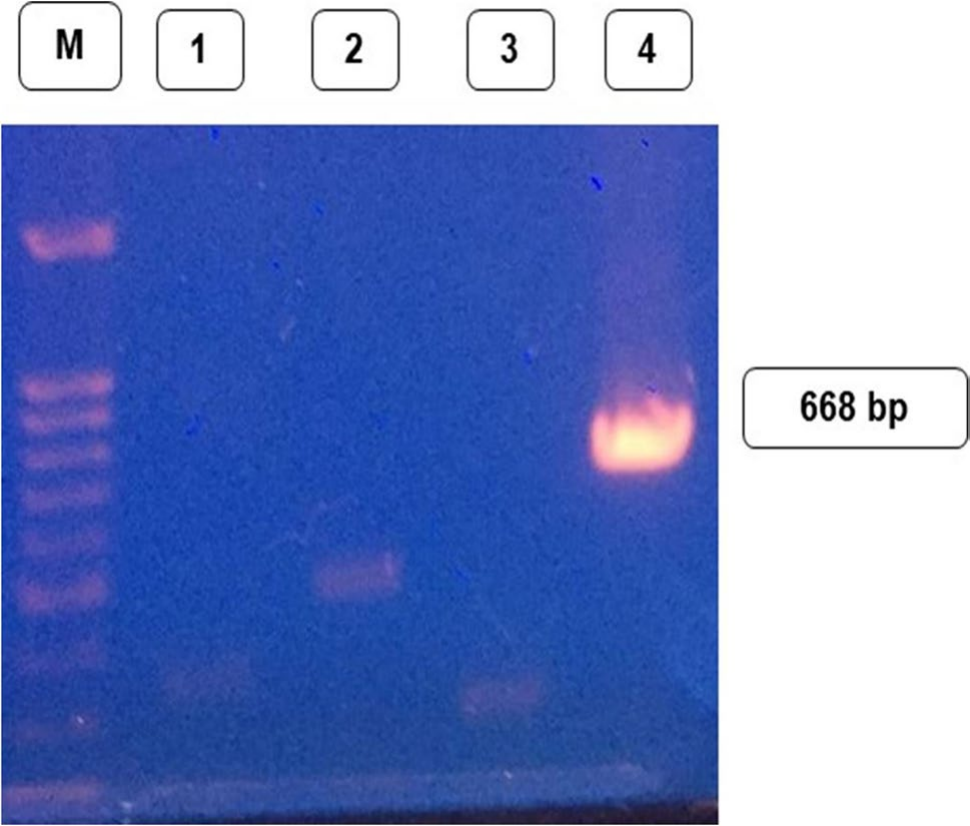
Testing the four primer sets with a positive control avian infection bronchitis vaccine. The primer pair PanCoV- F2 and PanCoV- R1 (lane 5) showed the clear remarkable band at the expected size 668 bp

The optimization of primers with the positive control showed that the primers that target a 668-fragment (PanCoV- F2 and PanCoV- R1) provided a clear band on expected size (Fig. 1).

**Fig. 2:**
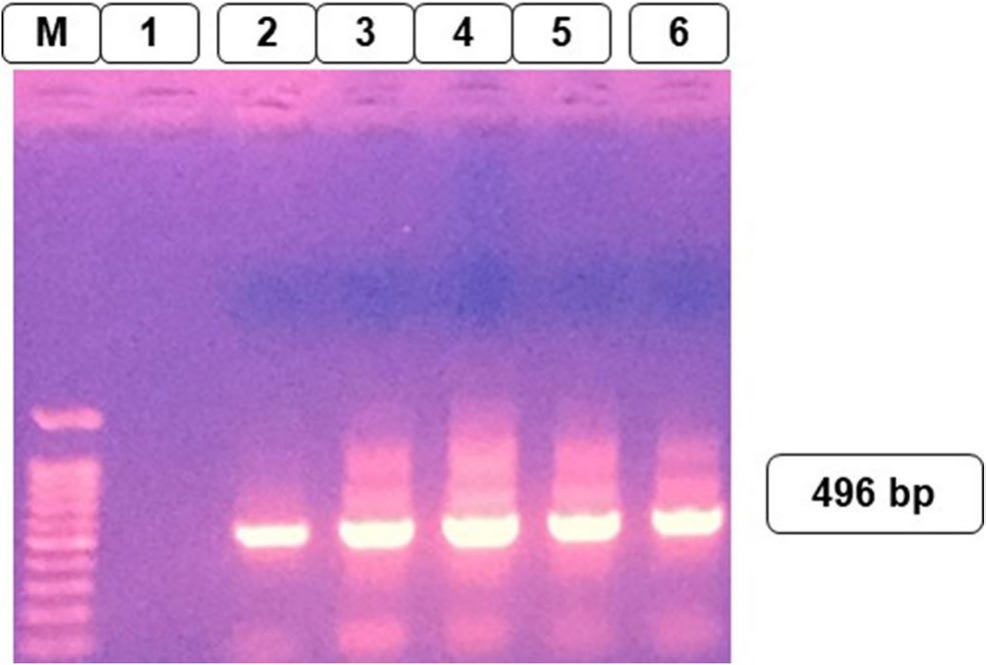
Amplification of GADPH (internal control) from RNA of human and bat samples. The first three positive bands are human samples, and the last two positive bands are bat samples. The bands are found at the expected size 496 bp

**Fig. 3:**
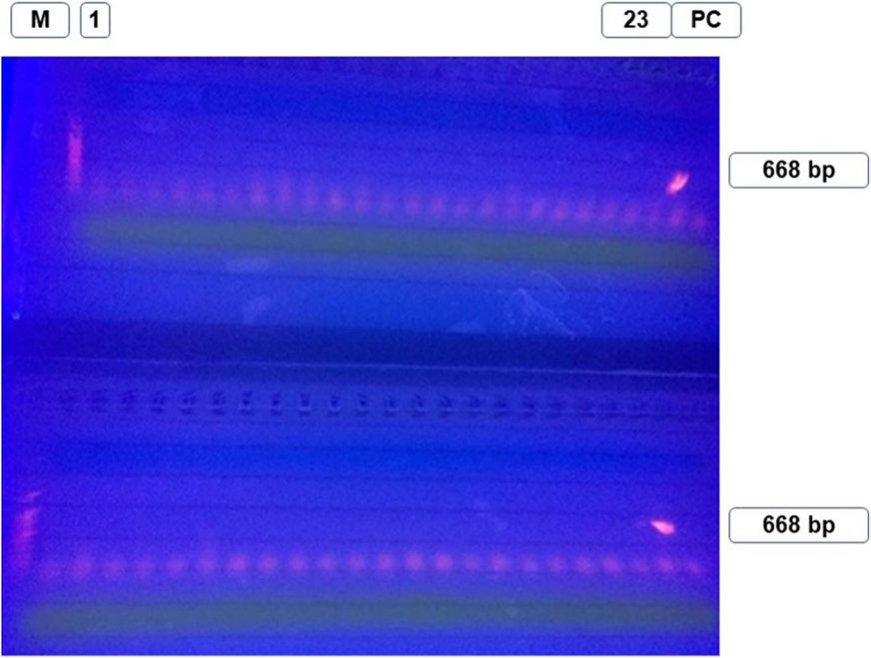
Bat samples (lanes 1–23 in each part) displayed no specific bands at the expected size 668 bp. Positive and negative controls are included in each part

To exclude the presence of PCR inhibitors in the samples, the extracted RNA from bats and human were tested by internal control primers (GADPH). All tested samples resulted in a clear single band of GADPH PCR product with the expected size (Fig. 2) which indicates integrity of RNA samples and feasibility of our PCR technique. RT-PCR analysis of all samples from bats (*Rousettus aegyptiacus* Geoffroy, 1810) for SARS–CoV-2 did not give any specific band at the expected size which indicates that these tested bat samples were free from corona virus infection (Fig. 3).

**Fig. 4:**
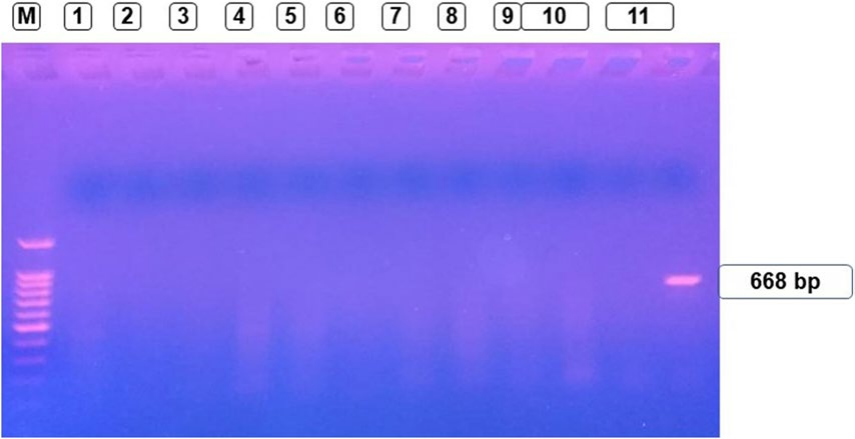
Dog samples (lanes 1–10) showed no specific bands at the expected size 668 bp. Negative control (11). Positive control is included

**Fig. 5:**
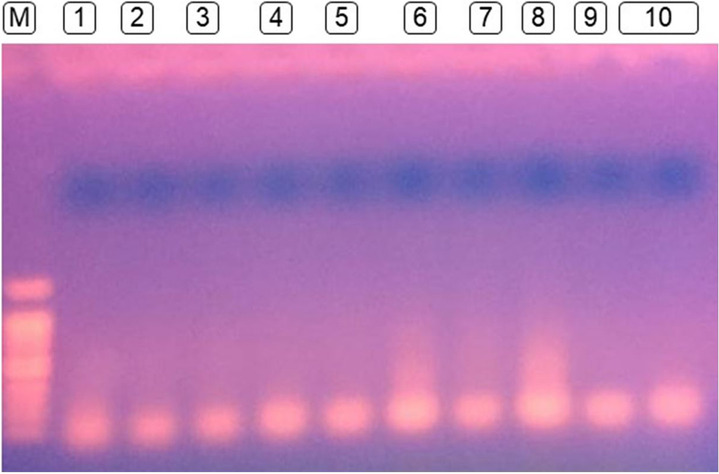
Migratory bird samples (lanes 1–10) showed no specific bands at the expected size 668 bp

Similarly, all tested samples from dogs, migratory and wild birds were negative to corona virus infection based on our RT-PCR results (Figs. 4 and 5, respectively).

**Fig. 6:**
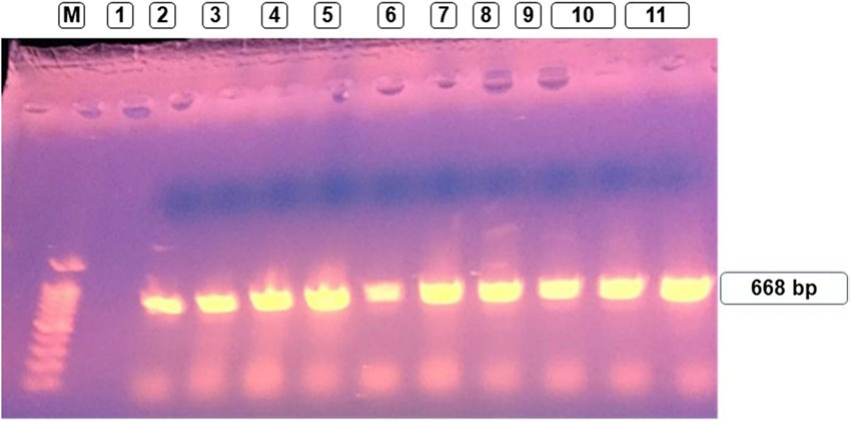
Human samples (lanes 2–11) showed specific bands at the expected size 668 bp

On the other hand, all samples collected from humans were RT-PCR positive to SARS–CoV-2 infection as they gave clear band, single band with the expected size (Fig. 6).

The results of Pan-corona RT-PCR in different animal species and humans are summarized in Table 3. To confirm that the obtained bands were specific to SARS–CoV-2, PCR products were subjected to sequencing and the obtained sequence data were analyzed using BioEdit and compared to the published GenBank database of RdRp gene of CoVs. Analysis of sequencing data confirmed that the obtained RT-PCR products are specific to SARS–CoV-2 which in turns confirmed that these individuals were positive to SARS–CoV-2 infection. The obtained SARS-CoV-2 RdRp nucleotide sequences are listed in Table 4. They showed high identity (≥ 99%) with SARS-CoV-2/human/USA/WA-CDC-UW21100279123/2021 and SARS-CoV-2/human/USA/AZ-ASU17288/2021 isolates.

**Table 3:** Summary of Pan-coronavirus RT-PCR results in different species

Name of species	Positive/total	Covs detected
Bats (*Rousettus aegyptiacus*)	0/46	None
Human	10/10	hCov-19/Egypt/USC- 1-6
Migratory birds (common teal, northern pintail and mallard)	0/20	None
Wild birds (swallow)	0/6	None
Pet animals (dogs)	0/10	None

**Table 4:** The SARS-Cov-2 RdRp nucleotide sequences obtained from human samples and their accession numbers

	Name of sequence	Accession No.
1	hCov-19/Egypt/USC-1/2021	EPI_ISL_8 464,608
2	hCov-19/Egypt/USC-2/2021	EPI_ISL_8 466,472
3	hCov-19/Egypt/USC-3/2021	EPI_ISL_8 469,597
4	hCov-19/Egypt/USC-4/2021	EPI_ISL_8 469,870
5	hCov-19/Egypt/USC-5/2021	EPI_ISL_8 470,239
6	hCov-19/Egypt/USC-6/2021	EPI_ISL_8 470,721

## Discussion

The role of animals in emergence and spread of the currently occurring SARS –CoV-2 pandemics still needs more investigation (Abdel-Moneim and Abdelwhab 2020; Frazzini et al. 2022). It is suspected that SARS-CoV-2 began in animals and was passed to humans, where it was subsequently spread from human to human (Ji et al. 2020). SARS-CoV-2 infection has been recorded in several animal species. The fact that SARS-CoV-2 can infect a wide range of animals suggests that the virus is capable of crossing the species barrier (Ji et al. 2020; Leroy et al. 2020). As a result, many wild and domestic animals may be infected with SARS-CoV-2 and serve as intermediate hosts for the virus (Tiwari et al. 2020; Wong et al. 2020; Zhao et al. 2020). The natural host of SARS-CoV-2 has been proposed to be the bat (Zhou et al. 2020). SARS-CoV-2 infection in cats, dogs, minks, tigers, and lions has been documented (Abdel-Moneim and Abdelwhab 2020; Oreshkova et al. 2020; Frazzini et al. 2022). In addition, natural infections of white-tailed deer, horse, and cattle have been recently documented (Kuchipudi et al. 2022; Pusterla et al. 2022; Wernike et al. 2022), and the list of animal species susceptible to infection continues to grow (Qiu et al. 2023). Moreover, mice, hamsters, cats, ferrets, non-human primates, and tree shrews, and goats have been shown to be susceptible to SARS-CoV-2 in experimental infections (Chan et al. 2020; Shi et al. 2020; Frazzini et al. 2022). The binding mechanism of SARS-CoV-2 RBD and ACE2 receptors has been studied structurally, and it appears that ACE2 from fish, amphibians, birds, and mammals can bind to SARS-CoV-2 RBD, making them potential natural hosts for SARS-CoV-2 (Chen et al. 2020). On the other hand, an initial study used a SARS-CoV-2 ELISA kit to identify SARS-CoV-2-specific antibodies in blood samples from 35 different animal species (Deng et al. 2020). Serum was collected from poultry (chicken, duck, and goose), experimental animals (mice, rat, and rhesus monkey), companion animals (cat and dog), domestic animals (sheep, pig, horse, and cow), and wild animals (leopard cat, masked civet, mink, ferret, jackal, fox, alpaca, camel, eagle, bamboo rat, peacock, tiger rhinoceros, porcupine, bear, giant panda, red pandas, pangolin, weasel, yellow-throated marten, and wild boar). There were no SARS-CoV-2-specific antibodies in any of the blood samples tested, ruling out the idea of these animal species serving as intermediate hosts for SARS-CoV-2 (Deng et al. 2020).

In the current study, we attempted to clarify the involving of animals in spread and transmission of SARS –CoV-2 among Egyptians. RT-PCR could be a useful, rapid and of reasonable cost tool for screening of the virus infection in different populations of animal species. The primers of the used PCR assay target the RNA dependent RNA polymerase gene of CoVs. The polymerase gene is a much-conserved region in the coronavirus genome. Therefore, the percentage sequence similarity will be high among different coronavirus members belonging to the same group. Nevertheless, this sequence information allows a primary identification of the coronavirus type that is present in a given sample (Vijgen et al. 2008).

Despite the numbers of individual animals sampled per species were relatively low (Table 1), our results revealed that no evidence for circulating of SARS –CoV-2 among animal populations in Egypt. Depending on the host and pathogen species, viral prevalence might vary significantly. We could have increased our detection rate in the species where no CoVs were discovered by targeting more host species and using bigger sample sizes. The same diagnostic technique, on the other hand, was able to detect all probable clinical cases in people. These findings could support the hypothesis that the animals at the present time are not the original source for infection for the human and most animal cases have had known or suspected exposure to human COVID-19 patients, indicating that human-to-animal infection is the primary cause of spread among domestic animals. The live-wild animal markets not famous in Egypt, therefore the possibility of inter-species contact between wild and domestic animal species is very low. Hence, the possibility of inter-species transmission of CoV infections needs to be confirmed by future studies, and these studies are required to understand if and how different animals could be affected by SARS-CoV-2.

Although the current available data of SARS-CoV-2 experimental infection in poultry suggest that poultry are not susceptible to SARS-CoV-2 infection and that the virus cannot be transmitted between humans and poultry (Frazzini et al. 2022), our study targeted migratory birds for searching for SARS-CoV-2 for following reasons; first, migratory birds act as hosts for a number of zoonotic viruses, and have the ability to disperse these viruses to distant geographic locations (Hepojoki et al. 2017). Second, CoVs represent a family of zoonotic viruses with wide variety of animal hosts, including birds and humans, and wild birds seem to serve as reservoirs for a variety of CoV strains (Velavan and Meyer 2020).

To summarize, basic hygiene precautions should be used to limit contact between sick people and animals, and in particular pets. Several animal models have been proposed for evaluating the effectiveness and safety of antiviral medicines as well as testing investigational vaccinations against SARS-CoV-2. To limit the spread of SARS-CoV-2, coordinated activities from several disciplines such as public health, veterinary medicine, environmental sciences, and social sciences are critical. These investigations will aid in the understanding of the virus’s possible hosts, the process of transmission, and the creation of vaccines. Furthermore, public health measures for workers who work with animals and animal byproducts are suggested, in addition to the use of basic hygiene procedures.

## Data Availability

All data generated or analyzed during this study are included in this published article. The nucleotide sequences are deposited in GISAID EpiCov database.

## Declarations

All animal handling procedures as well as samples collection and disposal were carried out according to the regulations of the Institutional Animal Care and Use Committee (IACUC), Faculty of Veterinary Medicine, University of Sadat City (Ethical approval number: VUSC-019-1-20).

The human samples were randomly selected from the left over samples in the laboratory that is working for diagnosis of COVID-19 for the community without any information about the patients.
